# Annual Research Review: Not just a small adult brain: understanding later neurodevelopment through imaging the neonatal brain

**DOI:** 10.1111/jcpp.12838

**Published:** 2017-11-03

**Authors:** Dafnis Batalle, A. David Edwards, Jonathan O'Muircheartaigh

**Affiliations:** ^1^ Centre for the Developing Brain School of Imaging Sciences & Biomedical Engineering King's College London London UK; ^2^ Department of Neuroimaging Institute of Psychiatry, Psychology and Neuroscience King's College London London UK

**Keywords:** Prematurity, perinatal, neurodevelopmental disorders, neuroimaging, biomarkers

## Abstract

**Background:**

There has been a recent proliferation in neuroimaging research focusing on brain development in the prenatal, neonatal and very early childhood brain. Early brain injury and preterm birth are associated with increased risk of neurodevelopmental disorders, indicating the importance of this early period for later outcome.

**Scope and methodology:**

Although using a wide range of different methodologies and investigating diverse samples, the common aim of many of these studies has been to both track normative development and investigate deviations in this development to predict behavioural, cognitive and neurological function in childhood. Here we review structural and functional neuroimaging studies investigating the developing brain. We focus on practical and technical complexities of studying this early age range and discuss how neuroimaging techniques have been successfully applied to investigate later neurodevelopmental outcome.

**Conclusions:**

Neuroimaging markers of later outcome still have surprisingly low predictive power and their specificity to individual neurodevelopmental disorders is still under question. However, the field is still young, and substantial challenges to both acquiring and modeling neonatal data are being met.

## Abstract 2

Read the Commentary on this article at doi: 10.1111/jcpp.12890

## Introduction

Research focusing on the perinatal and early postnatal period of human brain development has undergone rapid growth in recent years. Human brain structural and functional development occurs over a protracted period compared to many other mammals and primates (Watson, Desesso, Hurtt, & Cappon, 2006). This coordinated development provides the architecture for the expansion of behavioural and cognitive abilities, especially rapid in the first years (Johnson, 2001). In clinical neuroscience and paediatrics especially, there are clear reasons to focus on the early developing brain. The increasing survival of children born preterm has been a positive success but has the downstream consequences of an increased prevalence of neurological (Marlow, Wolke, Bracewell, & Samara, 2005), behavioural (Johnson et al., 2010) and cognitive difficulties (Saigal & Doyle, 2008).

An increased prevalence of neurodevelopmental disorders [such as autism spectrum disorder (ASD), subtypes of attention deficit hyperactivity disorder (ADHD) and later psychiatric illness] in children and adults born preterm (Johnson & Marlow, 2014) emphasises the importance and sensitivity of interruption during this period on later life outcomes. While these increased risks are widely reported, their anatomical and neurodevelopmental basis remain unclear.

From genetics, investigations of the underpinnings of neurodevelopmental diseases have begun to triangulate on families of genes active during early cortical patterning, associated with the establishment and maintenance of neuronal connectivity (Parikshak, Gandal, & Geschwind, 2015) or with the foundation of inhibitory/excitatory balance in early childhood (Marín, 2012). Importantly, findings are rarely disease specific, with genes predisposing to multiple different neurodevelopmental disorders (such as ASD and ADHD) and epilepsies (Zhu, Need, Petrovski, & Goldstein, 2014). However, the implication of these findings is that early markers of later disease may be observable prior to clinical phenotypic expression. For example in ASD, this has led to the search for behavioural, imaging and electrophysiological markers of atypical development in the infant brain (Elsabbagh & Johnson, 2010).

Even in the context of a clear lesion in the perinatal brain, it is extremely difficult to predict later cognitive or behavioural outcome in individual infants with a clinically useful accuracy. Heterogeneity in the clinical disorders themselves, in the developmental environment, as well as individual differences in resilience continue to cloud this search. With these challenges in mind, recent years have seen a proliferation of different techniques that allow us to quantify in vivo brain development at unprecedented spatial resolutions and biological specificity, putatively providing mechanistic insight into typical and atypical human development and, importantly, providing a bridge between animal models and human observations. This review will focus on novel techniques in brain imaging acquisition and analysis and discuss how their use in the pre‐ and postnatal brain has provided new insight into later cognitive and behavioural outcome in the typical and atypical brain.

## What happens during development and what can we see?

The perinatal period is a time of establishment, development and consolidation of brain connectivity. Important processes occurring during the neonatal period include the consolidation of thalamocortical connections and the continuous development of axons, with commissural and long‐range association fibres leaving the subplate and extending into the cortical plate (Kostovic & Jovanov‐Milosevic, 2006). Most developmental processes extend into the postnatal period, especially myelination, synaptogenesis and the formation of dendrites and dendritic spines, i.e. processes associated with interneuron *connectivity* (Kostovic & Jovanov‐Milosevic, 2006). Although macroscale white matter connections and long‐range inter‐ and intrahemispheric projections are established prenatally, short‐range cortico‐cortical connections continue to develop until at least the fourth month after birth (Burkhalter, 1993). Rapid dendritic arborisation and synaptogenesis results in an exuberance of connectivity after birth (Huttenlocher & Dabholkar, 1997; Innocenti & Price, 2005; Kostovic & Jovanov‐Milosevic, 2006), followed by a refinement of connections through pruning, selective elimination of dendrites and reshaping of axons, spanning later childhood and adolescence (Innocenti & Price, 2005).

Although most cortical neurons are generated by mid‐foetal life (Kjær, Fabricius, Sigaard, & Pakkenberg, 2017), there is ongoing postnatal migration of inhibitory neurons in the first few years of life (Paredes et al., 2016). However, the absolute number is limited and relatively small so most of the volumetric expansion of peri‐ and postnatal brain tissue is driven by astrocytes and oligodendrocytes (Kjær et al., 2017). *Post mortem* studies have charted postnatal neural cell proliferation, the linked process of myelination (Brody, Kinney, Kloman, & Gilles, 1987), synaptic proliferation and elimination (Huttenlocher & Dabholkar, 1997), dendritic sprouting and pruning (Petanjek et al., 2011), and illustrated the relative protraction of human postnatal development compared to our nearest primate relatives (Dobbing & Sands, 1979). However, *post mortem* studies are limited with respect to the degree to which we can characterise typical or atypical variation. While such studies can indicate the shape of developmental trajectories, they are not appropriate measures to quantify normative development. Noninvasive imaging, and particularly magnetic resonance imaging (MRI), has provided the opportunity to replicate classic and recent *post mortem* studies but, more importantly, to investigate longitudinally and in vivo the influence of individual differences in early brain structure and function on later outcome as well as their prognostic value in disease and injury.

## Imaging in the developing brain

**Figure 1:**
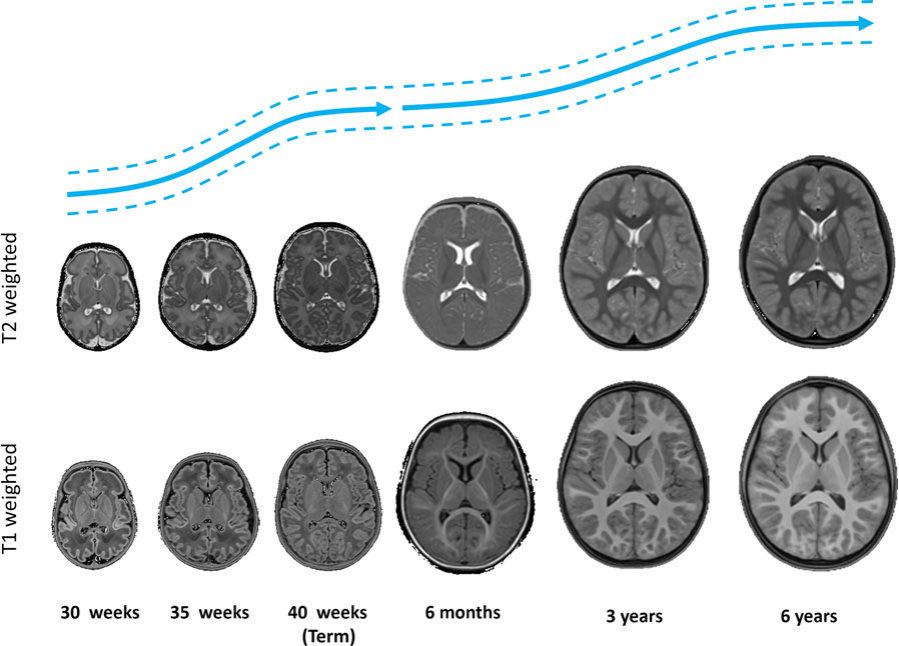
T1‐ and T2‐weighted images at different age points throughout the perinatal and early childhood period. Illustrative growth curves for the two periods (adapted from Makropoulos et al., 2016 and Dean III, Dirks et al., 2014) are shown above in blue [Colour figure can be viewed at http://wileyonlinelibrary.com]

In the past decade, there has been a clear improvement in the quality and resolution achievable with MRI, which has become the gold standard to study structural alterations as well as brain function and connectivity that can lead to altered neurodevelopment (Ment, Hirtz, & Huppi, 2009). However, both practical and technical aspects of MRI in the early period challenge established protocols usually devised for imaging the adult brain, emphasising an obvious point: the neonatal brain is not just a small adult brain. The cellular and connectional processes defining this period are associated with rapidly changing image contrast, tissue volume, and tissue morphology (Rutherford et al., 2006; see Figure 1). MRI scanners and protocols designed with adult measurements in mind are therefore suboptimal and do not consider the specific clinical needs of preterm neonates and infants. Fortunately, this also is rapidly changing. For the preterm‐born or at‐risk neonate, MRI‐compatible incubators (e.g. Erberich, Friedlich, Seri, Nelson, & Blüml, 2003) are widely available commercially. Specialist head coils have been designed with the infant's head in mind, increasing neonatal image signal‐to‐noise compared to equivalent adult head coils (Hughes et al., 2017; Scheef et al., 2017). Protocols have been published describing the practical aspects of scanning and preparation in both typical and atypical neonates and young children (Dean III, Dirks et al., 2014; Nordahl et al., 2016).

### Dealing with motion: The everyday in perinatal imaging

In terms of acquisition, even subtle intra‐ and interscan motion has been shown to severely compromise image fidelity in childhood and clinical cohorts (Power, Schlaggar, & Petersen, 2015). To account for this, sequences designed for compliant, still, awake adults are being redesigned from the bottom up to allow faster (Smith‐Collins, Luyt, Heep, & Kauppinen, 2015) or quieter imaging (Solana, Menini, Sacolick, Hehn, & Wiesinger, 2016), though not yet both. Novel approaches to motion correction using both prospective (Kuperman et al., 2011) and retrospective techniques have increased the success of structural (Cordero‐Grande et al., 2016) and functional/diffusion data acquisition in difficult samples (Maziero et al., 2016). These approaches are particularly relevant in the case of foetal imaging, where motion is extreme. In addition to allowing an even earlier understanding of the subtle brain changes in utero that lead to impaired neurodevelopment (Rutherford et al., 2008), foetal MRI acts as a useful testbed for the development of new sequences and postprocessing techniques that often are translatable to improve neonatal or even adult acquisitions (Jiang et al., 2007). In this scenario, there has been a notable improvement in the development of optimised acquisition sequences (Hayat et al., 2011; Malamateniou et al., 2011) and improved volume reconstruction and postprocessing of motion artefacts (Fogtmann et al., 2014; Jiang et al., 2007; Kuklisova‐Murgasova, Quaghebeur, Rutherford, Hajnal, & Schnabel, 2012). These developments have allowed the assessment of accurate volumetric, tissue and shape characteristics of both the foetal and neonatal brain (e.g. (Rajagopalan et al., 2011; Wright et al., 2014).

## Measuring cortical shape and tissue content

### T1 and T2 during development

Typical MRI techniques rely on the spin‐lattice relaxation time (T1) or spin–spin relaxation time (T2) in order to obtain an anatomical map of the tissue under study. Given that different tissues have different magnetic properties (relaxation times) dependent on the tissue environment and microstructure, they produce tissue‐specific contrast in different areas of the brain and at different ages (see Figure 1). Development of the neonatal brain is fast, rapidly changing the tissue relaxation characteristics observable with MRI (Deoni, Dean, O'Muircheartaigh, Dirks, & Jerskey, 2012; Kulikova, Hertz‐Pannier, Dehaene‐Lambertz, Poupon, & Dubois, 2016; Leppert et al., 2009). This contrast maturation combines with morphological changes including rapid increases of volume (Knickmeyer et al., 2008), gyrification patterns (Dubois et al., 2008), and cortical thickness (Nie et al., 2014), making image analysis over this period extremely challenging.

These challenges have necessitated the construction and adaptation of age‐appropriate brain templates and atlases to much tighter age‐ranges (e.g. weekly Makropoulos et al., 2014) compared to childhood (monthly or annually, e.g. Deoni et al., 2012; Fonov et al., 2011). These atlases specifically adapted to the developing brain have eased the arduous task of manually delineating regions and tissue types (Gousias et al., 2013; Kuklisova‐Murgasova et al., 2011; Shi et al., 2011). In addition, a natural extension of age‐specific atlases has been the creation of *continuous* atlases of brain development, characterised by formal models of image intensity, tissue position and tissue volume (Dean III et al., 2015; Kuklisova‐Murgasova et al., 2011; Kyriakopoulou et al., 2016; Makropoulos et al., 2016). These models in particular have provided the opportunity to develop regional normative ‘charts’ of development throughout the brain, allowing the calculation of percentiles (Holland et al., 2014), akin to head circumference charts.

### MR tissue relaxometry: The promise of real quantification

Due to this changing contrast over development, an attractive acquisition option for structural imaging is MR tissue relaxometry (Deoni, 2010). In place of acquiring a single contrast image with fixed parameters, a series of weighted images (e.g. T1, T2, T2*) are collected with an aim to calculating quantitative tissue relaxation times, which should be constant at a given MR scanner field strength, but regionally variable, characteristic of local tissue properties. The clear disadvantage of these multi‐image approaches is a relatively long acquisition time compared to a standard single‐weighted anatomical image acquisition at the same resolution. Therefore, there is an increase in motion sensitivity, but there still are good reasons why this area has been a recent focus in the developing brain especially. First, intersite variability *should* decrease (Deoni et al., 2008; Weiskopf et al., 2013), and, at least, intersite differences can be better quantified. The relaxation rate images can be used to create *synthetic* weighted images with optimum contrast for the tissue or age of interest (Krauss, Gunnarsson, Andersson, & Thunberg, 2015). This means that a single acquisition scheme can be used for an entire population, but different weighted contrasts could be generated for each individual, appropriate to their age or the pathology being investigated.

These parameters can also have a biological interpretation, especially when used in combination (Weiskopf et al., 2013). Given an appropriate model, relaxometry can be used to profile different water pools within a unit voxel, with the different profiles presumed to reflect different tissue water environments, and, of particular interest, myelin (Kolind, Mädler, Fischer, Li, & Mackay, 2009). T2 relaxation, for example, shows a rapid decrease in white matter over the first two postnatal years (Leppert et al., 2009), reflecting the changes in tissue contrast mentioned earlier and long known observations from postmortem measurement of increases in myelin content (Brody et al., 1987). This decrease in T2 values has been associated directly with an increase in myelin‐associated water content (Deoni et al., 2012; Kulikova et al., 2016).

## Quantifying tissue architecture

### Diffusion MRI

Like relaxometry, diffusion MRI is also a quantitative technique, but in addition to providing information on tissue water content, it allows a measurement of the restriction of water diffusion. Models of the restriction of water diffusion can be used to provide estimates of the dominant direction and anisotropy of water diffusion and, from this, reconstruct presumed white matter bundles. Notwithstanding several limitations (Tournier, Mori, & Leemans, 2011), the most commonly used model to date has been diffusion tensor imaging (DTI). Since its inception, it has been applied to study the rapid changes in white matter microstructure during neonatal development (Counsell et al., 2003; Huppi et al., 1998), mainly using measures such as mean diffusivity (MD) and fractional anisotropy (FA) to characterise apparent water diffusion and tissue organisation respectively. In the developing brain, MD values of the white matter typically decrease with age, with FA increasing, consistent with the decrease of water content (Dobbing & Sands, 1973), and increase in oligodendrocyte and myelin (Yakovlev & Lecours, 1967) processes occurring early in brain development (Huppi & Dubois, 2006). Although typically applied to study white matter, DTI has also shown promise in assessing grey matter development, showing a *decrease* in FA values as the brain approaches term age (Ball, Srinivasan et al., 2013), consistent with arborisation processes during early development in cortical tissue (McKinstry et al., 2002). These changes are region dependent, with primary sensory and motor cortices developing at different rates to association cortices, for example, mirroring in grey matter microstructure what is seen in the underlying myelination patterns in white matter (Dean et al., 2015).

### Beyond the tensor: Advanced diffusion MRI

As an alternative to this standard tensor model, advanced computational methods have been proposed that allow the association of tissue characteristics to the diffusion MRI signal in a more data‐driven fashion. The diffusion tensor is a relatively simple model that fails in the context of multiple fibre populations. This can be addressed by using high angular resolution diffusion imaging (HARDI), and techniques such as diffusion spectrum imaging (Hagmann, 2005), Q‐Ball (Tuch, 2004) or constrained spherical deconvolution (CSD; Dell'Acqua et al., 2007; Tournier, Calamante, & Connelly, 2007). All are capable of reporting multiple fibre orientations in a given voxel and provide a more realistic reconstruction of underlying structure governing the diffusion signal. These advances have allowed us to obtain neuroanatomically accurate reconstructions of white matter tracts that were impossible to delineate with tensor approaches. They have permitted, for instance, the delineation of complex white matter tracts such as the arcuate fasciculi (Salvan et al., 2017) and cerebello‐cerebral connections in neonates (Pieterman et al., 2017) as well as the development of technically challenging *post mortem* atlases of foetal white matter tract bundles (Takahashi, Folkerth, Galaburda, & Grant, 2012).

The assessment of microstructural characteristics in these tracts has great potential to provide anatomically grounded and clinically relevant associations with later neurodevelopment. As well as more accurately delineating white matter bundles and structures, new multicompartment models can provide appropriate estimations of microstructural characteristics such as neurite density and orientation dispersion in vivo (Jespersen, Kroenke, Ostergaard, Ackerman, & Yablonskiy, 2007; Zhang, Schneider, Wheeler‐Kingshott, & Alexander, 2012), providing distinct information to measures like FA and MD. Particularly, the use of neurite orientation and density imaging (NODDI) model is becoming increasingly popular, and is starting to be applied to study more accurately microstructural changes occurring in the developing brain, in both white matter (Kunz et al., 2014) and grey matter (Eaton‐Rosen et al., 2015). Recently, novel techniques have been proposed to also obtain quantitative estimations of fibre *density* that are becoming both more reliable and computationally feasible (Pestilli, Yeatman, Rokem, Kay, & Wandell, 2014; Smith, Tournier, Calamante, & Connelly, 2013). Combined with accurate estimation of white matter microstructure (e.g. with NODDI), they represent a powerful approach to characterise the emergence of anatomical connectivity during development (Batalle et al., 2017). Making use of both quantitative estimates of tissue architecture (e.g. NODDI) and quantitative estimate of myelin content (using relaxometry), two groups have tried to approximate the g‐ratio of white matter, the ratio of internal axon diameter to myelinated axon diameter. This measure of axonal conduction efficiency (Rushton, 1951) reduces longitudinally from high levels (around 0.9, indicating very little myelination) in the preterm thalamus to 0.85 at approximately term age (Melbourne et al., 2016) and continues to decrease over the first few years of postnatal life in the rest of white matter (Dean III et al., 2016).

## Assessing tissue function

### Neonatal functional MRI: A technical challenge

This changing conduction efficiency, wiring and structure, underlies the progressive development of cortical and subcortical function. The blood oxygenation level dependent (BOLD) contrast is the most commonly used MRI measure of brain function. As haemoglobin has different magnetic properties when bound to oxygen, it acts as an endogenous contrast agent with good contrast to noise and spatial specificity when measured using T2*‐weighted sequences. By repeatedly measuring BOLD signal over a period of time, relative increases and decreases of the signal can be interpreted as changes in activity (Logothetis, Pauls, Augath, Trinath, & Oeltermann, 2001). In general, an experimental perturbation, such as a sensory stimulus or cognitive/emotional elicitation (Donaldson & Buckner, 2001), is provided to the participant in a time‐locked fashion, and the BOLD response to this, a convolution of the haemodynamic response function and the experimental design, is statistically modelled throughout the brain.

**Figure 2:**
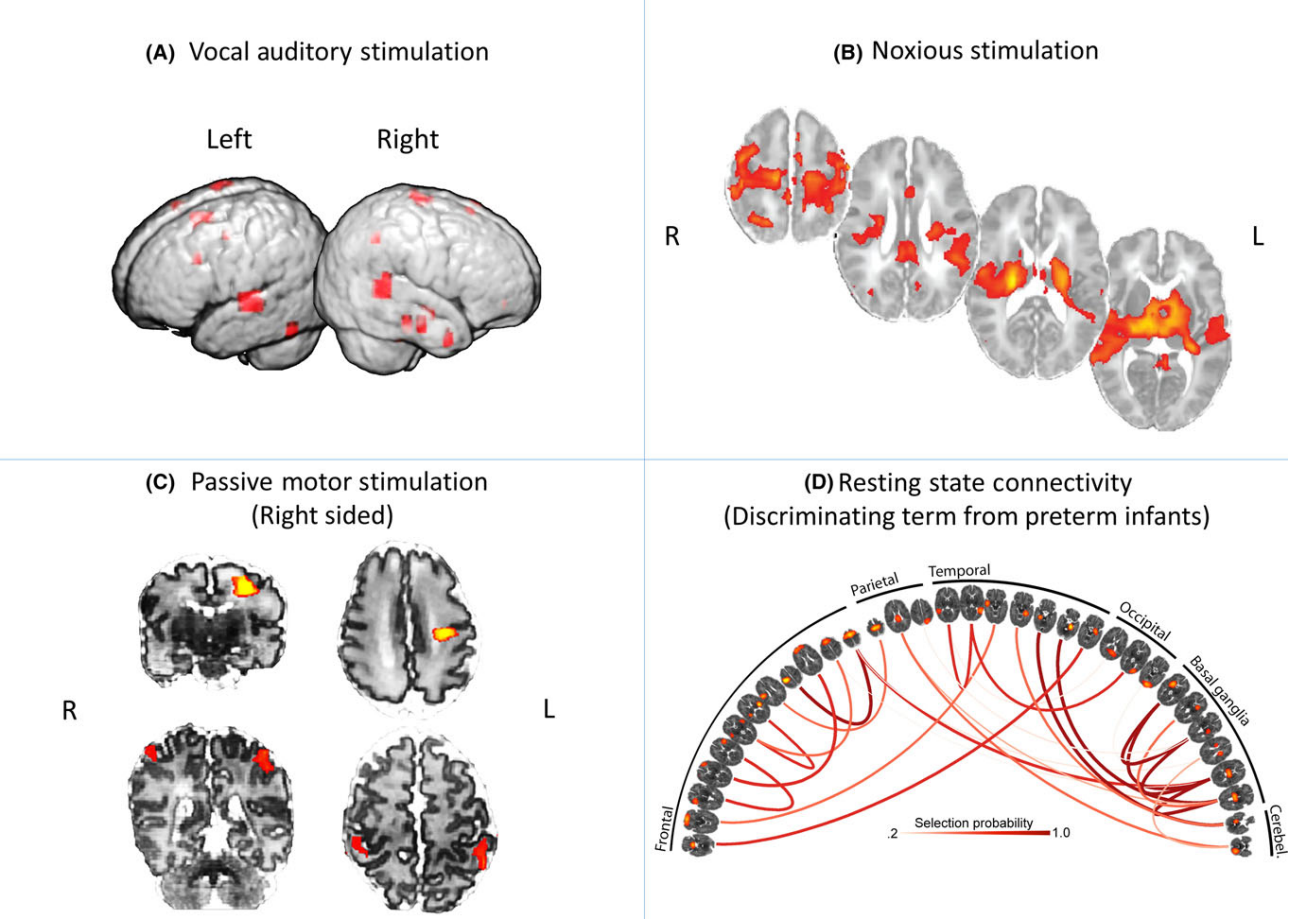
Functional MRI applied to the neonatal brain in both task‐based and resting conditions. For auditory stimulation, provided during natural sleep, adult‐like patterns of voice‐responsive activity become evident (Blasi et al., 2015), (A). Similarly, the activity patterns in response to painful skin stimulation reflects what would be seen in adults (Goksan et al., 2015), (B). Motor cortex responses to a passive motor balloon task become more bilateral from 31 weeks (top row, C) to term age (41 weeks, bottom row, C) (Arichi et al., 2012). Finally the repertoire of resting state networks seen in adults is clearly evident in the neonatal brain at term‐equivalent age (Ball et al., 2016), (D), but the pattern of connectivity between the networks is different in the preterm‐born brain, allowing discrimination [Colour figure can be viewed at http://wileyonlinelibrary.com]

Obviously, the assessment of brain activity in response to specific tasks and conditions in experimental paradigms designed for awake, responding adults, presents limitations when studying the brain of a sleeping, sedated or even awake neonate. To address this substantial practical problem, innovative solutions and experimental designs have been proposed, focusing on auditory, visual, tactile and olfactory stimuli (e.g. Arichi et al., 2010; Dehaene‐Lambertz et al., 2010; Erberich et al., 2003; Goksan et al., 2015). For example, an experimental paradigm presenting voice and nonvoice auditory stimuli to the sleeping infant demonstrated voice selective cortical regions in the temporal lobe (Blasi et al., 2015; Figure 2a). Although these results have tended to show adult‐like *spatial* patterns (see Figure 2), the amplitude and even the direction of the BOLD response (e.g. positive or negative association of BOLD signal) were inconsistent in early studies (Seghier, Lazeyras, & Huppi, 2006). Importantly, these earlier inconsistencies could be explained by observed differences in the haemodynamic response function (HRF) in neonates. Specifically, the HRF shows an age‐dependent reduction of its time to peak, a change in the poststimulus under‐shoot and a general increase in amplitude from 32 to 46 weeks postmenstrual age (PMA; Arichi et al., 2012), highlighting the necessity to use neonatal‐matched HRFs in task‐based functional MRI (fMRI) studies (Goksan et al., 2015).

### Functional MRI of babies ‘at rest’

With few exceptions, task‐based fMRI studies in neonates tend to focus on sensory stimuli or receptive language (passive listening). This understandable bias limits our knowledge of functional networks that are known to be associated with higher cognitive functions and goal related behaviour. An alternative to task‐elicited functional activation is to look at spontaneous fluctuations of this same signal. The formative study of Biswal, Yetkin, Haughton, & Hyde (1995) demonstrated low‐frequency fluctuations in the resting brain with a high degree of temporal correlation and predictable anatomical structure, suggesting its association with actual functional connectivity. This work has been extended substantially in the intervening years, with the investigation of the physiological and cognitive relevance of these low‐frequency fluctuations near dominating recent cognitive and clinical neuroimaging research. Importantly, the brain networks delineated extend beyond somatosensory, with cortical–subcortical networks that reflect the full range of task‐elicited functional networks but in the absence of an explicit task (Smith et al., 2009). For these reasons, resting state fMRI has been leapt on in neonatal imaging and it has been used to demonstrate and map the functional organisation of the healthy neonatal brain. Using independent component analysis (ICA) to filter and isolate coherent fluctuations, resting state fMRI BOLD signals exhibited resting state networks spatially matching some of those previously described in the adult brain in studies of cross‐sectional term and preterm infants, during light sedation and natural sleep (Fransson et al., 2009). Both ICA and seed‐based correlation approaches have also been used in longitudinal studies of preterm and term infants showing the emergence of connections partially or completely matching several resting state networks (RSN) during neonatal development (see Figure 2d), including the default mode network (Doria et al., 2010; Gao et al., 2009; Smyser et al., 2010) and detailed thalamocortical connections resembling those seen in adults (Toulmin et al., 2015).

### In utero functional MRI: The next frontier?

In parallel with neonatal fMRI, foetal fMRI has developed enormously during recent years, including pioneering comprehensive pipelines that integrate rapid acquisitions and post hoc correction of motion artefacts (Ferrazzi et al., 2014, 2016). This approach shows great promise for understanding the different patterns of in utero functional brain connectivity as compared to premature *ex utero* environmental experience (Thomason et al., 2017). In addition, it provides the opportunity to further develop early biomarkers of altered neurodevelopment based in MRI, as early as the third trimester of pregnancy. This area is still under active technical development. Features of the foetal and neonatal brain, especially the prolonged T2*, mean that standard fMRI sequences will need to be adapted and optimised (Ferrazzi et al., 2016).

## The developing brain as a network

**Figure 3:**
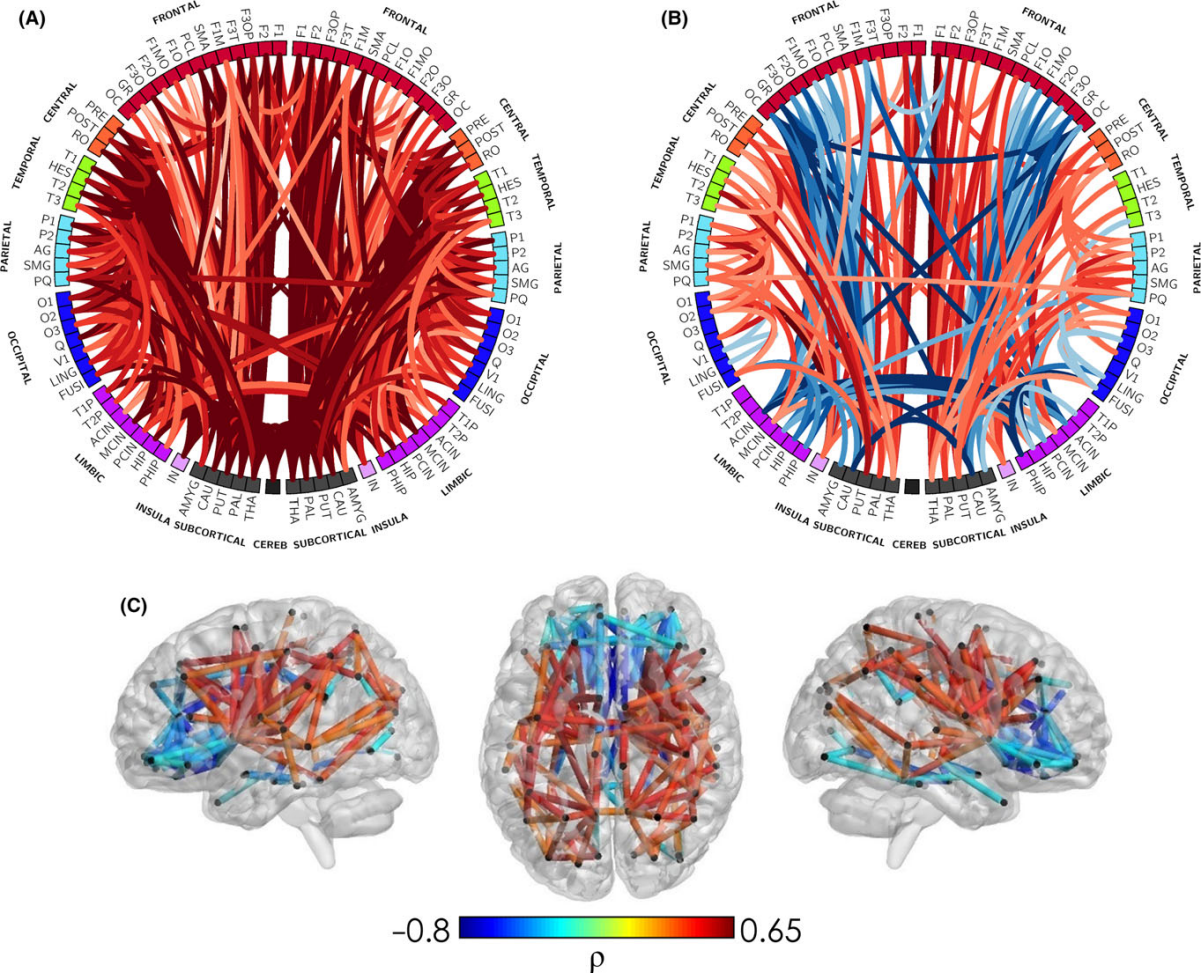
Network representation of developing microstructure. The correlation of corrected age at MRI with structural connections weighted by NODDI intracellular volume fraction index (or neurite density index, NDI), in a population of 65 infants scanned between 25 and 45 weeks of postmenstrual age (PMA). In panel (A) NDI parameters increase with age for most white matter connections, as would be expected. However, when assessed in relative terms (%) of the total connectivity in each subject (panel B and C), it is possible to separate which connections are developing at a relative faster or slower pace. This clarifies the expected heterochronicity in the early development of brain connectivity, with a general trend of connections between somatosensory, central, subcortical and temporal areas to show faster development (red) than frontolimbic and interhemispheric connections (blue). Adapted from Batalle et al. (2017) [Colour figure can be viewed at http://wileyonlinelibrary.com]

Individual brain regions do not function in isolation, but act in coherent, anatomically constrained networks. Both explicitly and implicitly, fMRI tends to be focused on describing these networks but an approach that is proving successful in development is the use of MRI to investigate properties of this network structure. Network approaches (see Figure 3) chart attributes of ‘nodes’ (usually brain regions) and their pattern of either structural or functional connections (‘edges’, typically calculated using diffusion or functional MRI respectively). The assessment of the structural macroscopic connectivity of the brain obtained from the reconstruction of white matter tracts with tractography based in diffusion MRI, has been used extensively to study brain connectivity in health and disease (Bassett & Bullmore, 2009).

### Complex networks for (complex) developing brains

Since the initial investigations of this approach in human data, there has been great interest in tracking the emergence of brain connectivity both in typical and atypical brain network development and in finding the association between subtle alterations in connectivity and altered neurodevelopment. In the context of broader MRI research, one of the great advantages of network techniques is that the use of network paradigms allows a reduction in the dimensionality of brain connectivity to just a few comprehensible features that represent key characteristics of network infrastructure and organisation (Rubinov & Sporns, 2009). In particular, the global organisation of brain networks can be characterised using three main graph theoretical concepts: infrastructure (e.g. network density and average strength), integration (e.g. global efficiency or characteristic path length) and segregation (e.g. local efficiency or average clustering coefficient). During early development, macrostructural connectivity has been characterised using structural network models based in diffusion MRI showing an increase in both integration capacity and segregation properties (Brown et al., 2014; van den Heuvel et al., 2015). These changes are coherent with changes in type and number of connections, as the relative proportion of short‐ and long‐range association fibres changes during brain maturation (Huang & Vasung, 2014). Other findings include small‐world characteristics (van den Heuvel et al., 2015; Tymofiyeva et al., 2012) showing high segregation and integration properties and asymmetries between hemispheres in graph theoretical measures during development (Ratnarajah et al., 2013), both consistent with findings in the adult brain. A ‘rich club’ of regions (van den Heuvel & Sporns, 2011), so called because they are highly connected to each other, has also been demonstrated in preterm subjects, as early as at 30 weeks of gestational age (Ball et al., 2014), also coherent with adult findings indicating the early establishment of these topological features.

Studies considering graph theoretical parameters of *functional* brain networks of the neonatal brain are less common. The emergence of cortical functional hubs and small‐world characteristics in the neonatal brain was first shown by Gao et al. (2011) while voxel‐wise networks obtained in a normalised space were obtained by Fransson, Aden, Blennow, & Lagercrantz (2011), demonstrating the presence of cortical hubs and their associated subnetworks. More recently, the feasibility of assessing dynamic functional connectivity (i.e. the way in which functional network connectivity changes over scanning time) as well as models of synchronisation, have also been demonstrated in the neonatal brain (Batalle et al., 2016). Extending these unimodal models, van den Heuvel et al. (2015) assessed functional and structural networks concurrently in a cross‐sectional sample, showing a relatively high level of structural–functional coupling that increases with age. Although it is obvious that structural and functional connectivity are intrinsically linked (Honey, Thivierge, & Sporns, 2010), the study of their interface may help us to better understand, in addition to the structural characteristics underpinning brain function, subtle alterations in the organisation of connectivity that may have a dramatic effect in functional activity, and that could lead to an atypical developmental trajectory (Deco & Kringelbach, 2014). This approach can help understand how functional connectivity during development is driven by the structural rich club of highly connected nodes (Ball et al., 2014), and the functional implications of connections not belonging to this rich club, which have been reported to be altered in response to preterm birth (Ball et al., 2014; Batalle et al., 2017).

### Mapping anatomical similarity: Morphologically based brain networks

Diffusion and functional MRI are the current standard approaches to infer connectivity using MRI. However, the availability of conventional anatomical T1/T2 acquisitions in clinical practice has led to approaches to infer connectivity based only on anatomical features. Given that the sensitivity of diffusion and functional MRI to motion artefacts in foetal and neonatal groups, as well as their relative rarity, this approach is especially relevant in development. Based on the concept that correlations of grey matter features such as volume or cortical thickness across *groups* of individuals are associated with brain connectivity (He, Chen, & Evans, 2007), anatomical T1 acquisitions have been used to obtain group brain networks that allow a better understanding of brain circuitry in neuropsychiatric and neurodegenerative disorders (Bassett et al., 2008), and the characterisation of those networks during early development (Fan et al., 2011). However, to develop *individual* predictors of altered neurodevelopment, it is necessary to extract brain connectivity at an individual level, which typically is not possible with single observations of tissue volume. A number of published reports have suggested alternative approaches to extract individual brain networks based on the analysis of the similarities of grey matter features (Raj, Mueller, Young, Laxer, & Weiner, 2010; Tijms, Series, Willshaw, & Lawrie, 2012). To which extent these networks resemble anatomical brain networks is an issue that remains to be elucidated, but reports suggest that cytoarchitectonic similarity reflect anatomical brain connectivity (Beul, Barbas, & Hilgetag, 2017). They have shown potential to be associated with early neurodevelopment (Batalle et al., 2013) and hence, to become an alternative tool to analyse and detect abnormal cortical patterns where the acquisition of diffusion and/or functional MRI presents clear challenges, as is the case of foetuses and neonates.

## The effect of early adversity – Imaging the brain at risk

### Imaging the preterm brain

Preterm birth, the interruption of normal in utero gestation prior to term age, is common; approximately 11% of all live births worldwide are prior to term (Blencowe et al., 2012), with rates mostly higher in developing compared to developed countries. The substantial neurological, behavioural and cognitive comorbidity associated with prematurity means that improving our understanding of both the altered neurodevelopment and its long‐term effects (Nosarti et al., 2012) is one of the primary challenges of modern clinical developmental neuroscience. With this in mind, neuroimaging has been widely used to assess how the preterm‐born infant's brain deviates from the patterns of typical development.

Conventional structural MRI of severe preterm babies detects a high prevalence of diffuse excessive high signal intensity in white matter observed in T2‐weighted images (Maalouf et al., 1999), associated with more diffuse white matter abnormalities using diffusion MRI (Counsell et al., 2003) and adverse neurodevelopmental outcome (Boardman et al., 2010). Further quantitative assessment of diffusion MRI in neonates has demonstrated that alterations of the white matter are also present in preterm babies without visible focal lesions on clinical MRI (Anjari et al., 2007; Huppi et al., 1998) and, again, even these very subtle brain abnormalities are associated with altered neurodevelopmental performance at 2 years of age (Counsell et al., 2008). Other studies have also shown the impact of prematurity on several aspects of brain morphology in neonates, including volumetric changes in the cerebellum (Limperopoulos, Chilingaryan, Guizard, Robertson, & du Plessis, 2010), cortical and subcortical grey matter (Padilla, Alexandrou, Blennow, Lagercrantz, & Aden, 2015; Tolsa et al., 2004), and an altered shape of the hippocampus, with volume associated with verbal memory during childhood (Thompson et al., 2013). These changes are relevant to the cognitive outcome of these children. Alterations in the structural characteristics of the arcuate fasciculus of preterm babies scanned at term have been shown to correlate with later language development at 2 years (Salvan et al., 2017), mirroring results seen later on in preterm‐born adolescents (Mullen et al., 2011). Thalamocortical connectivity, one of the later connectional processes occurring at mid to late term period, has also been shown to be different in preterm infants at term‐equivalent age (Ball et al., 2012) and associated with impaired cognitive performance at 2 years of age (Ball et al., 2015). Complementing these observations, functional connectivity strength between cortical areas and thalamic nuclei are also significantly reduced in preterm subjects (Toulmin et al., 2015). Although alterations in connectivity explain part of the variance in later cognitive outcome, clinical and environmental aspects, especially socioeconomic status, explain a larger part (Ball et al., 2015).

### Pathoconnectomics of the preterm infant

It is still not clear if alterations in connectivity (‘pathoconnectomics’) are a sufficient phenotype to define or delineate neuropsychiatric disorders (Rubinov & Bullmore, 2013). Nonetheless, this approach has undoubtedly brought some understanding on the systems mechanisms that might be associated with specific alterations in cognition and behaviour in adulthood (Crossley et al., 2014). In the context of perinatal conditions, it provides the opportunity to assess if there are early markers of alterations in connectivity associated with atypical development. This would go in line with the perinatal origin hypothesis of neurodevelopmental disorders as ASD and schizophrenia. By using graphs to model macrostructural brain connectivity, brain networks obtained from diffusion MRI tractography have been used to demonstrate that preterm babies present a pattern of reduced cortical and subcortical connectivity (Batalle et al., 2017) that is also present at 1 year of age (Pandit et al., 2014). Furthermore, the rich club organisation of structural brain networks during the preterm period shows a relative preservation of core connectivity in preterm babies at term‐equivalent age (Ball et al., 2014), continuing to school‐age (Fischi‐Gomez et al., 2016) and adulthood (Karolis et al., 2016). Similar findings have been reported in preterm‐born children during the perinatal period, but using NODDI to weight connectivity by microstructural parameters of the white matter tracts connecting brain cortical regions. In this case, in addition to a conserved core of connections, peripheral connections showed reduced neurite density indices (Batalle et al., 2017).

Changes in the distribution of microstructural weights affect network topology as measured by graph theory characteristics, which establishes for the first time a systems mechanism underpinning neurodevelopmental alterations associated with preterm birth. So brain networks and graph theoretical analyses show great potential to assess subtle connectivity changes, though interpretation is not straightforward, and must be considered thoroughly (Van den Heuvel et al., 2017). Interestingly, the effective use of scarce white matter resources (Karolis et al., 2016) is coherent with the developmental origin of mental disorders (Gluckman, Hanson, & Beedle, 2007), at a systems neuroscience level. Adverse perinatal events might affect the global capacity to establish typical connectivity patterns, leading to changes in typical myelinogenesis, but possibly also inducing a certain level of reorganisation of the structural network topology. It seems a plausible hypothesis then, that in the presence of a shortage of energetic resources or other adverse perinatal events, some developmental neuronal processes subtly shift their preference to ensure core, vital brain connectivity, even at the cost of alterations in connections associated with important aspects of cognition and behaviour, including executive function, memory, attention, information processing, response inhibition, salience processing and emotion regulation.

### Intrauterine growth restriction

When compared with prematurity, intrauterine growth restriction (IUGR) is a perinatal condition with clearer aetiology. Associated with placental insufficiency, IUGR affects 5‐10% of all pregnancies and it is a leading cause of foetal morbidity and mortality (Jarvis et al., 2003). Reduction of placental blood flow results in sustained exposure to hypoxaemia and undernutrition (Baschat, 2014) and has severe consequences on the developing brain (Rees, Harding, & Walker, 2011), including neurodevelopmental and cognitive dysfunction during the neonatal period (Bassan et al., 2011; Figueras et al., 2009), childhood (Eixarch et al., 2008; Feldman & Eidelman, 2006; Geva, Eshel, Leitner, Valevski, & Harel, 2006) and early adulthood (Løhaugen et al., 2013). Hence, there has been a considerable effort to characterise the underlying brain alterations supporting these dysfunctions and the prediction of the subset of the population with a higher risk of altered neurodevelopmental outcomes. MRI has been used to study brain alterations associated with IUGR in utero, showing deeper sulcation measurements in the insula and cingulate fissure (Egaña‐Ugrinovic, Sanz‐Cortes, Figueras, Bargallo, & Gratacos, 2013) and different texture patterns associated with neonatal behaviour (Sanz‐Cortes et al., 2013). Interestingly, during the neonatal period, an altered pattern of gyrification in IUGR babies has also been described and associated with later altered neurodevelopment (Dubois et al., 2008). While the effects of IUGR in brain network organisation and its association with altered neurodevelopment have been extensively assessed in later infancy and childhood (e.g. Batalle et al., 2012; Munoz‐Moreno et al., 2016), its assessment in newborns remains scarce. It has been suggested that functional connectivity is already altered at term in newborns with IUGR (Batalle et al., 2016), which is consistent with hyperconnectivity found in ICA networks at 1 year of age (Padilla, Fransson et al., 2017). However, the interaction between functional and structural connectivity in this population remains unclear, as does its capacity to predict babies *at risk* of neurodevelopmental disabilities at birth.

### Other perinatal conditions: Isolated ventriculomegaly and congenital heart disease

Perinatal conditions with lower prevalence are also a focus of perinatal neuroimaging research, as they serve as an example of how different brain alterations are linked with altered neurodevelopment. Even when presented without other associated comorbidities, ventriculomegaly has been linked with a further increased risk of developing neuropsychiatric disorders (Gilmore et al., 1998) and developmental difficulties (Leitner et al., 2009). Foetal MRI studies show in utero cortical overgrowth (Kyriakopoulou et al., 2014), reduced FA and increased MD values in the white matter in subjects that later show a higher risk of language delay (Lockwood Estrin et al., 2016). In neonates, isolated ventriculomegaly has been shown to be associated with reduced FA values, specifically in the splenium of the corpus callosum, as well as an increase of MD in other white matter tracts (Gilmore et al., 2008). Congenital heart disease (CHD) is the most common congenital malformation, affecting almost 1% of live births (Hoffman & Kaplan, 2002), and has been associated with behavioural problems and neurodevelopmental delays during childhood (Bellinger et al., 2009). Hence, CHD has become a growth area of research in the recent years, being linked with increased prevalence of white matter injury, lower FA in the white matter (Miller et al., 2007), altered metabolism (Dimitropoulos et al., 2013) as well as cortical immaturity (Clouchoux et al., 2013) associated with altered neurodevelopment at 2 years of age (Beca et al., 2013). Recently, Birca et al. (2016) have shown an interesting association between microstructural brain abnormalities and functional connectivity measured with EEG in patients with CHD. However, further studies are required in order to thoroughly characterise brain connectivity in these infants, and to determine whether there is a pattern of atypical early connectivity that can predict later altered neurodevelopment.

### MRI and ultrasound: A practical partnership

Although our focus here is neuroimaging using MRI, this excludes by far the most common technique for foetal and neonatal imaging. Ultrasound is the clinical workhorse in pre‐ and perinatal imaging and has been the technique of choice to study in utero development. It has been also widely applied to study abnormal development in the neonatal brain, taking advantage of the acoustic windows present in the newborn skull, typically the anterior fontanelle. Cranial ultrasound remains the modality of choice for bed‐side neuroimaging in the neonatal intensive care unit to detect brain damage, including haemorrhage, ventriculomegaly, lenticulostriate vasculopathy and some congenital malformations, being predictive of any major lesion found in MRI during childhood (Rademaker et al., 2005). It is affordable, widely available, and relatively easy to use and house. This is especially important in rural areas and developing countries where other imaging techniques such as MRI might be difficult or impossible to access. However, in order to become a useful diagnostic tool for subtle brain damage, and a screening tool for altered neurodevelopment, is essential to fully utilise the richness of data accessible with this technique by computational methods that go beyond what is observable by the naked eye (Tenorio et al., 2014; Vansteenkiste, Huysmans, Govaert, Lequin, & Philips, 2008). To make this effort successful, wider and larger studies that correlate ultrasound findings with underlying alterations in white matter structure such as those observed on MRI are paramount. Importantly, abnormalities observed on neonatal ultrasound including periventricular haemorrhage and periventricular haemorrhage with ventricular dilation are associated with later cognitive and behavioural problems during adolescence (Nosarti et al., 2011), and with white matter alterations in memory‐related tracts measured with diffusion MRI tractography in young adults, which scored lower in tests assessing visuospatial memory and efficient organisation of information (Caldinelli et al., 2017). Early studies have assessed correlations between neonatal ultrasound and MRI (e.g. Leijser et al., 2009; Maalouf et al., 2001;de Vries, Benders, & Groenendaal, 2013); however, more detailed associations of ultrasound findings with neurological measures from MRI could enhance the use of ultrasound as a screening tool of altered neurodevelopment, with MRI as gold standard. This link is especially relevant in everyday clinical practice, as improving the capacity of neurosonography to predict neurocognitive outcome has the potential of making a real difference for screening babies at risk.

## Neurodevelopmental disorders in their infancy

### Assessing the perinatal risk factors of ASD

The neurological and neurodevelopmental sequelae, such as ADHD and autism, that can be associated with such a broad range of perinatal conditions strongly indicate that this period is an important time to understand both idiopathic and genetic forms of these disorders. Broadly, neurodevelopmental disorders have been conceptualised as ‘anomalies in neurodevelopmental trajectories’ (Shaw, Gogtay, & Rapoport, 2010) or ‘adaptive variants of typical trajectories’ (Elsabbagh & Johnson, 2010). In this context, prematurity is one of many biological or environmental insults that could push the trajectory of the developing brain to an atypical path. In autism for example, neuroimaging results have been largely consistent with this concept of multiple atypical trajectories. Initial observations of head circumference differences in late infancy/early childhood who later develop autism (e.g. Courchesne, Carper, & Akshoomoff, 2003) indicate that some autisms may be indicated by atypical brain growth in early life. Although these and other similar findings have been challenged on appropriate use of population norms (Raznahan et al., 2013) as well as limited reproducibility (Zwaigenbaum et al., 2014), they have nonetheless spurred the investigation of the early developing brain in infants considered at high genetic risk (due to a sibling with the condition), providing the opportunity to investigate the disorder prior to any treatment exposure.

A series of reports from the Infant Brain Imaging Study (IBIS), the Autism Phenome Project (APP) and others, have focused on brain structure in infants at high risk of ASD due to a first‐degree sibling diagnosed with ASD. Interrogating structural MRI measures, these studies have reported early biomarkers of autism in infants observable prior to diagnosis. Using different analyses and samples, they may indicate converging approaches to subtyping aetiologically diverse ASDs. Focusing on grey matter and cortex, Hazlett et al. (2017) demonstrated that expansion of cortical surface area between 6 and 12 months could be used to predict later diagnosis with autism, again compared to other high‐risk infants, and that these changes preceded atypical longitudinal cortical volume expansion between 12 and 24 months. Parallel work from Ohta et al. (2016) similarly showed cortical surface increases in autism using a cross‐sectional cohort at 36 months. However, the age specificity of these putative biomarkers in autism seems to be marked. For instance, neither study showed changes in cortical thickness, something that seems to be evident in older cohorts (e.g. Ecker et al., 2013). Earlier work on IBIS cohort (Hazlett et al., 2012) was unable to detect cross‐sectional volumetric differences between 6‐month olds classified as at‐risk or not (though not separated according to later autism diagnosis as in their more recent analyses). The risk factor for later diagnosis also clearly matters to the putative marker as *reduced* cortical volumes in areas associated with social and salience integration have been reported in neonates surviving extreme prematurity and later diagnosed with ASD (Padilla, Eklof et al., 2017).

Prompted initially by an observation of increased incidental radiological findings in a group of high‐risk infants, Shen et al. (2013) have now twice demonstrated an increase in extra‐axial CSF volume in infants later diagnosed with autism. Interestingly this increase scaled with symptom severity and negatively correlated with motor abilities (Shen et al., 2017), providing a replication to their previous work but in independent samples (again from the IBIS consortium). They were also able to demonstrate how cerebral volume and CSF volume independently and significantly contribute to head circumference values, providing a direct link to earlier work. This marker is also seen in adolescents and older adults with autism (Hallahan et al., 2009), although changes in total brain volume do not tend to be evident by that age.

### Complex analyses for complex disorders

A feature of these studies is that they have used relatively simple measures of brain structure (cortical volume, CSF volume, cortical area). As detailed earlier, graph theoretic measures have widely been applied in the developing brain, showing sensitivity to both prematurity and postnatal maturity, but these measures have not yet been shown to be very sensitive to atypical development in neurodevelopmental disorders. In older toddlers, Dinstein et al. (2011) showed atypical resting‐state interhemispheric connectivity in auditory cortex in children diagnosed with autism. Importantly for specificity, this connectivity change was distinct from children with developmental language delay. Another study from the IBIS consortium looked at functional connectivity from a large range of infants at both low and high risk for autism (aged 6 and 12 months). Analysing whole‐brain connectivity of these infants, they could successfully classify infant age using support vector machines, but a classifier designed to separate infants based on familial risk of ASD was unsuccessful (Pruett Jr et al., 2015). Graph theoretical measures, calculated using connectivity obtained from diffusion MRI, did show subtle reductions in global efficiency in 24‐month‐old infants with a later diagnosis of ASD (Lewis et al., 2014), and the same measures were shown to be present at an earlier age (6 months) in the same cohort in more recent work (Lewis et al., 2017). In this way, summary measures of brain connectivity, like global efficiency, appear to be consistent markers of atypical development, at least in early childhood. Investigating groups at‐risk for neurodevelopmental disorders in early life is still a recent phenomenon and the use of advanced analysis approaches (such as network analysis) is more recent still.

### From neurodevelopmental ‘risk’ to pathological phenotypes

Although promising, most of the studies assessing early markers of autism have the weakness of focusing on an ‘at‐risk’ subgroup of children, being unclear whether these results map to this subgroup specifically (e.g. those at genetic risk) or to the broader autistic phenotype. When looking at increased head circumference as a biomarker, it is important to note that macrocephaly *and* microcephaly can contribute to the diverse autism aetiology (Libero, Nordahl, Li, & Amaral, 2017; Raznahan et al., 2013) and this kind of heterogeneity likely reduces consistency between brain studies. The specificity of these results to autism should also be challenged further. The two studies by Shen and colleagues show differences in the later diagnosed ASD infants that partly mirror results seen in, for example, male infants at increased genetic risk of schizophrenia (Gilmore et al., 2010).

This brings up a wider if not obvious point. Neurodevelopmental disorders are aetiologically diverse. They represent families of pathological phenotypes and are commonly comorbid with each other. A good rationale for investigating infants who later develop autism is that neuronal architecture abnormalities have been noted on tissue pathology reports and these abnormalities occur in utero. So, for example, malformations of cortical development such as heterotopias, focal cortical dysplasias, or similarly spatially circumscribed patches of disordered cortical layering, have been described postmortem in a growing set of autism studies (Casanova et al., 2013; Stoner et al., 2014). However, these neuropathological markers, associated with abnormalities of radial or tangential neuronal migration during early or late embryonic development, are similar to those more commonly detected after surgical resection in focal epilepsies, especially in childhood (Blümcke, Sarnat, & Coras, 2015). In single gene mutations associated with autism, there tends to be significant comorbidity with other disorders including epilepsy but also schizophrenia, ADHD and intellectual disability (Jeste & Geschwind, 2014). Many research studies on autism, for example, exclude participants with a history of seizures, leading to a further bias in the literature and ignoring what could be a useful endophenotype (Berg & Dobyns, 2015).

### Understanding heterogeneity: Big data in development

As has been discussed in this forum before (Zhao & Castellanos, 2016), the categorical nature of investigations of behavioural and cognitive neurodevelopmental disorders may well hinder both genetic and neuroimaging studies of atypical development. In this context, novel approaches trying to disentangle the heterogeneity of neurodevelopment and neurodevelopmental diseases in a data‐driven and symptom‐driven way (e.g. Marquand, Wolfers, Mennes, Buitelaar, & Beckmann, 2016) should be welcomed. Cohort studies investigating increasingly large cohorts of developing neonates and children (the developing Human Connectome Project (dHCP), the NIH baby Connectome project, the UNC early brain development studies, the IBIS cohorts, the Autism Phenome Project, the ePrime study of preterm brain imaging) are providing large and well‐characterised populations of healthy children. The follow‐up of these children in the coming years may provide sufficient numbers to allow us to start to split coarse diagnostic categories by following the natural history of disease development (as in Fountain, Winter, & Bearman, 2012). Although the numbers of individual studies are very large by neuroimaging standards (all studies mentioned have acquired or have a target sample size of >500), they are small in comparison to samples seen in genetics. These resources have therefore been augmented by automated retrospective and prospective data sharing through schemes like the National Database for Autism Research (NDAR https://ndar.nih.gov/). The developing Human Connectome Project (dHCP http://www.developingconnectome.org/) and the upcoming Baby Connectome Project (BCP http://www.thevirtualbrain.org) will also share data prospectively.

Where big data may also have a large positive influence is in the domain of imaging genetics; the association between population genetic variability and imaging phenotypes. The massively multivariable nature of both data types mean that large sample sizes are critical. Prior work has successfully focused on a subset of risk genes that are associated with adult psychiatric disorders to investigate their influence on early brain development (as in Dean III, Jerskey et al., 2014; Knickmeyer et al., 2014; Qiu et al., 2015). However, this approach is limited to a strong a priori hypothesis as to disease pathogenesis and genetic effect or, in the absence of strong a priori hypothesis, vast sample sizes (e.g. the ENIGMA consortium Franke et al., 2016). As is the trend in adult cohorts (Dima & Breen, 2015), studies in infants are instead beginning to make use of the polygenic nature of complex diseases explicitly in study designs. In the case of a polygenic risk score for example, genetic susceptibility for disease is assumed to be a cumulative influence of weak contributions from many variants and these cumulative risk scores are then used as a continuous predictor of brain structure. Exploratory analyses using this approach have just begun to arise, for example investigating how polygenic risk scores for major depression, derived from an independent population study, can interact with an environmental factor (in this case maternal depression) to influence the infant brain (Qiu et al., 2017).

## From making observations to explaining behaviour

### Predicting outcomes from inherently confounded data

This is not easy. Detecting real effects, true links between brain imaging and behaviour or disease, is especially difficult in the developing brain. Normal maturational changes in brain structure and function are orders of magnitude larger than individual differences. Although an average difference of a year or more between two groups of adults is unlikely to have a large effect on detection sensitivity when investigating disease, in the neonate and throughout the first years of life, an average separation of a month would likely occlude or confound any real effects of interest. Although major developmental pathologies such as chronic lung disease and hypoxic ischaemic encephalopathy have been shown to induce large amplitude effects on white matter (changes of 5%–15%, Ball, Boardman et al., 2013), neurodevelopmental disorders and, by definition, subtle abnormalities of prematurity, will not be so evident. In studies not looking at disease or pathology, where the interest is in individual differences in cognitive abilities or developmental function, and again where large effect sizes would not be expected (Gignac & Szodorai, 2016), the need for very large sample sizes becomes more obviously apparent.

### Machine learning as a solution?

Multivariate ‘pattern classification’ approaches to investigation may provide a partial solution to this problem. Resulting multivariate patterns can represent larger effect sizes relative to standard voxel‐wise univariate analyses (Reddan, Lindquist, & Wager, 2017). The downside (or upside depending on your perspective) is that methods such as pattern classification can be agnostic to the mechanism that gives the diagnostic or predictive pattern; the provided biomarker or pattern does not need to be biologically interpretable (Castellanos, di Martino, Craddock, Mehta, & Milham, 2013). As machine learning techniques become more highly nonlinear (e.g. in deep learning applications), human interpretation of the discriminating model becomes very difficult (Ravì et al., 2017).

As opposed to statistical association that typically is trained and validated in the whole population available, machine learning is generally implemented by training a model in a subsample of the data available that links input data (e.g. neuroimaging features) and output values (e.g. neurodevelopmental characteristics). Then, the validity and quality of the model is tested in previously unseen data sets, bringing value to the ‘true’ concept of *prediction*, instead of *association*, which is a more appropriate term to describe classical statistical fitting. In the field of machine learning, there are many methods to select relevant input characteristics and to find useful models, though a first approach is to use previous knowledge of the data to select relevant input features and models (e.g. alterations in the pyramidal tract may be associated with later motor outcome). Algorithms agnostic to previous knowledge are becoming very popular, having clear value for exploring new hypotheses, helping to elucidate links that may not be obvious. A downside is that this may come at the price of ignoring previously known mechanisms, leading to biologically implausible conclusions.

So, again, study design and sample selection are even more important in development. For binary classification problems (e.g. will someone have a good or poor outcome), a confounding difference in age of even a week, between two groups or treatments being classified, could drive classification accuracy. The application of machine learning approaches to neonatal data is still relatively novel. It has been used to better understand alterations associated with prematurity at birth, showing that term and preterm babies can be classified blindly based on their patterns of functional connectivity (Ball et al., 2016; see Figure 2D). Wee et al. (2017) explored the use of graph theoretical clustering coefficients of neonatal brain networks to group healthy babies with lower or higher emotional and behavioural scores at 2 and 4 years of age. They also used a machine learning approach to find the regions that contribute more to classify between those two groups. Kawahara et al. (2017) specifically adapted a convolutional neural network framework to structural brain networks and demonstrated the capacity to predict neurodevelopmental scores at 2 years of age based in structural brain networks at birth. Although showing a relatively modest predictive power (correlations up to .3), the approach of predicting continuous variables instead of dichotomised indices shows great potential. Importantly, in addition to the potential development of image biomarkers, machine learning approaches can be used to find relevant brain connections that contribute to later neurodevelopment. In this way, it may provide a new understanding of the mechanisms associated with brain development and provide potential markers of improvement in response to treatment.

## Building towards the future

### The power of mathematical abstraction

Crucial to improve predictive power and interpretability in machine learning is the use of appropriate neuroimaging features that are capable to describe subtle changes in brain connectivity underpinning atypical neurodevelopment. Network science is a promising framework to obtain such features, and is a focus of research in modern computational neuroscience. In recent years, there have been several advances in the way information can be extracted from brain networks. One approach is the use of generative models, i.e. mathematical rules that define how a realistic brain network can be generated based on a set of parameters. These parameters and mathematical rules are the *abstract* laws that model brain structure and potentially development. For instance, Betzel et al. (2015) have exhaustively studied generative models of brain networks, showing the importance of considering geometrical constraints of the distance between brain regions in order to appropriately generate such models. This would obviously be age‐dependent in early development. The physical location of brain areas is not random, but the result of a slow process of genetic programming and developmental sculpting, and even when constructing abstract models this information needs to be taken into account as inappropriate definition of nodes has been shown to greatly distort network metrics (Smith et al., 2011). Importantly, appropriate generative models have the potential to sensitively characterise mechanisms underlying normal and abnormal brain connectivity topology, and might be especially relevant when attempting to understand the emergence of human connectivity. Another interesting development in graph theory is the use of measures characterising information spreading dynamics, modelled as cooperative and competitive cascades, which can be a powerful method to understand the transitions between cognitive states (Misic et al., 2015). Additionally, the use of controllability theory brings light to how exogenous input might affect this spreading of information in brain network systems (Betzel, Gu, Medaglia, Pasqualetti, & Bassett, 2016). These systems neuroscience approaches involve a high level of mathematical abstraction. Nonetheless, if appropriately applied, the characterisation of brain network models with complex systems might have very real applications to detect subtle brain changes leading to altered behaviour and cognition. The application of such approaches has been scarce in perinatal neuroimaging, but initial studies investigating altered neurodevelopment with models that describe, for example, information spread, have shown great promise (Chung et al., 2016), and its wider application in neurodevelopment is warranted.

### The structure–function interface: Informing MRI with underlying biophysics

Models of structure–function coupling, accumulated from fields like electrophysiology and even particle physics, also have the potential to realistically describe macroscopic interactions in the brain. This approach may change the way neuroimaging data will be analysed in the near future, by providing a *virtualisation* of macrostructural connectivity that could be useful for studying brain development and subtle alterations leading to atypical behaviour and cognition (e.g. The Virtual Brain project: www.thevirtualbrain.org, Sanz‐Leon, Knock, Spiegler, & Jirsa, 2015). This virtualisation of the brain is performed by characterising the relationship between structure and function using models of spontaneous local neuronal activity. However, macroscopic neural activity is only weakly dependent on individual neuronal behaviour, and so can be described by mesoscopic top‐down approximations of brain complexity using mean field model reduction of the spiking neuron model (Deco et al., 2013), which simplifies enormously the computational load of such models. This is currently a very active area of research (Park & Friston, 2013) and there is hope that these kinds of approaches will deliver fundamental new insights on brain functional architecture (Sporns, 2013). These approaches allow in silico connectivity representations of the brain to provide biologically informed information, complementing current structural and functional connectivity techniques (van den Heuvel & Sporns, 2013). They show great potential for understanding altered brain dynamics associated with psychiatric and behavioural disorders (Deco & Kringelbach, 2014), and offer the possibility of not only enhancing our understanding of the emerging organisation of brain connectivity in the healthy infant but also elucidating alterations in brain connectivity associated with neurodevelopmental disorders.

Applying these models to the developing brain is challenging; it is not clear that states resembling resting state fMRI dynamic functional connectivity in adults (Hansen, Battaglia, Spiegler, Deco, & Jirsa, 2015) will be sufficient to explain functional and structural characteristics of the unmyelinated neonatal brain, and its characteristic and rapid plasticity. Indeed, the relationship between structure and function almost certainly changes over development (Hagmann et al., 2010), requiring the addition of a temporal dimension to test the versatility of the models developed. It is also unclear to what extent the apparent excitatory nature of GABA in the neonatal brain (Ben‐Ari, 2002) will require thorough modifications of the models used. Nonetheless, we expect that the wider application of computational models to characterise early brain development will be critical to improve our understanding of typical and atypical cognitive and behavioural development from neuroimaging, providing missing explanatory power from the association of structure to function.

## Conclusion

The current expansion in neonatal neuroimaging imaging research reflects an increasing awareness that many neurological and psychiatric diseases could be better described as disorders of brain development, with a perinatal origin. Although techniques are still being developed to analyse the very different MR characteristics of this challenging population, improvements are already evident, reflected by a shift in the field from exploratory to applied studies. Population studies of typical structural and functional brain development have yielded to investigations of genetic and environmental influences on individual differences in this development. Applying this to atypical neurodevelopment, brain connectivity measures have provided predictive value on later progression to complex disorders such as autism, and are beginning to provide individualised predictions on behavioural and cognitive outcome in childhood.

Enhancing explanatory power in such applied studies is critical. Classifiers and biomarkers derived from neonatal neuroimaging data have thus far been simple, rarely addressing more than one neurodevelopmental outcome, and have specificity values that would misdiagnose healthy children at a rate that would greatly outnumber the prevalence of the disorder in question. For instance, increased CSF may be a good biomarker of a poorer developmental outcome given a history of autism in the family, but it is unclear whether this would extend to children with autism but no family risk, or whether it precludes other developmental disorders. The large heterogeneity of both typical brain development and disorders of neurodevelopment would indicate that future investigations of neuroimaging markers of later outcome will become more complex, not less. This requirement for complex models will need to be balanced by the need for clinical markers to be interpretable and grounded in biology.

The rate of advances both within the field and outside indicate that challenges can be met. Both technical and practical advances in neuroimaging techniques have led to high‐throughput, high quality and publicly available data sets of the neonatal brain. Perinatal neuroimaging is increasingly a mature field, achieving image quality and sample sizes comparable to those seen in adults. This opens the opportunity to apply for the first time advanced techniques and models that were inconceivable until recently. There is great hope that by putting together these technological and analysis tools, we can shine light on the key neurodevelopmental processes underlying individual cognitive and behavioural development and provide sensitivity to the subtle brain alterations leading to their dysfunction.

**Key Points**

- The investigation of the neonatal brain provides an opportunity to study developmental processes that may influence both typical and atypical neurological and behavioural functioning in the child and adult.
- Neonatal neuroimaging is technically challenging, with standard adult pipelines of image acquisition and image processing confounded by the naturally changing tissue content, shape and size of the neonatal brain.
- Damage to specific anatomical and functional pathways, such as those from the thalamus to cortex, can begin to explain later neurodevelopment in infants born prior to term.
- Impressive recent work has started to demonstrate longitudinal infant brain markers of later‐onset childhood neurodevelopmental disorders, and especially autism.
- Further advances, particularly in machine learning and in computational models of the brain, might have the potential to improve our understanding of the link between the neonatal brain and the developing child.
